# Lessons From the Queensland Paediatric Cardiac Service Neurodevelopmental Follow-up Programme

**DOI:** 10.1016/j.cjcpc.2022.04.006

**Published:** 2022-05-27

**Authors:** Karen Eagleson, Robert Justo

**Affiliations:** a Queensland Paediatric Cardiac Service, Queensland Children’s Hospital, Children’s Hospital and Health Service, Brisbane, Queensland, Australia; b Faculty of Medicine, the University of Queensland, Brisbane, Queensland, Australia

With advances in surgical procedures and treatment, most children with even the most complex congenital heart disease (CHD) are now surviving into adulthood.^1^ As a result, there is now an increased focus on the associated neurodevelopmental morbidity resulting from a not yet fully understood interplay between clinical, genetic, and sociodemographic risk factors.^2^, ^3^, ^4^ Parental stress in families of children with CHD can impact their own wellbeing, the family environment, and subsequent development of the child.^5^ Although many will have normal development, infants and children with CHD can experience impacts across all developmental domains, with the emergence of new issues over time.^6^^,^^7^ These issues can impact schooling success, future employment, mental health, and quality of life.^2^^,^^6^^,^^8^

## Long-term Developmental Follow-up for Children With Congenital Heart Disease

In 2012, the American Heart Association (AHA) published a scientific statement^2^ providing recommendations for neurodevelopmental evaluation and management from infancy into adolescence, including periodic formalized assessment for children at highest risk, to support timely identification of delays and subsequent early intervention. More recently, expanded neurodevelopmental evaluation strategies for infants and children under 5 years of age^9^ and school-age children^10^ have been released through the Cardiac Neurodevelopmental Outcome Collaborative, an international organization that aims to establish best neurodevelopmental practices and improve outcomes for children with CHD and their families.

Since the release of the AHA recommendations, there has been an increase in formalized cardiac neurodevelopmental follow-up programmes as standard clinical care, predominantly across the United States. There is considerable variability in the programme structure, mode of service delivery, diagnosis and age eligibility, multidisciplinary team membership, and timing and nature of assessments.^11^, ^12^, ^13^, ^14^ Importantly, formalized follow-up programmes have resulted in the establishment of registries to facilitate longitudinal data collection and quality improvement. However, there is an identified need for evidence of the impact of these programmes on neurodevelopmental outcomes and what elements are of greatest value and importance to families.^11^^,^^15^ Further, challenges to programme development and attendance have been identified including high cost and resourcing, limited inclusion of school-age children, and travel implications for families to the surgical centre.^2^^,^^11^^,^^16^^,^^17^

## Long-term Developmental Follow-up for Children With Congenital Heart Disease—An Australian Context

Building on existing work and in response to the AHA recommendations, in 2013, the Queensland Paediatric Cardiac Service (QPCS) established the CHD Long-term Improvement in Functional eHealth (LIFE) programme to support the long-term developmental follow-up of children at high risk for poorer outcomes. A pilot centralized clinic located at the surgical centre evolved to a statewide decentralized approach, supporting access to developmental services close to home.^18^

Our early programme was established as a partnership between QPCS and the local child development service, including select high-risk cohorts (transposition of the great arteries, hypoplastic left heart complex, and post extracorporeal life support) with protocolized assessment of development, quality of life, and parenting stress informed by US-based programmes. The potential impact of geographical limitations to formalized developmental follow-up in Australian populations with CHD has previously been identified.^19^ Despite the large catchment area of QPCS, clinic attendance was consistently high. However, programme cost and resourcing challenges within a public health care system resulted in unsustainable service delivery, with recognition of the cost to external hospital and health services to support attendance of a centralized clinic, and to families when services are not delivered close to home.

**Figure 1:**
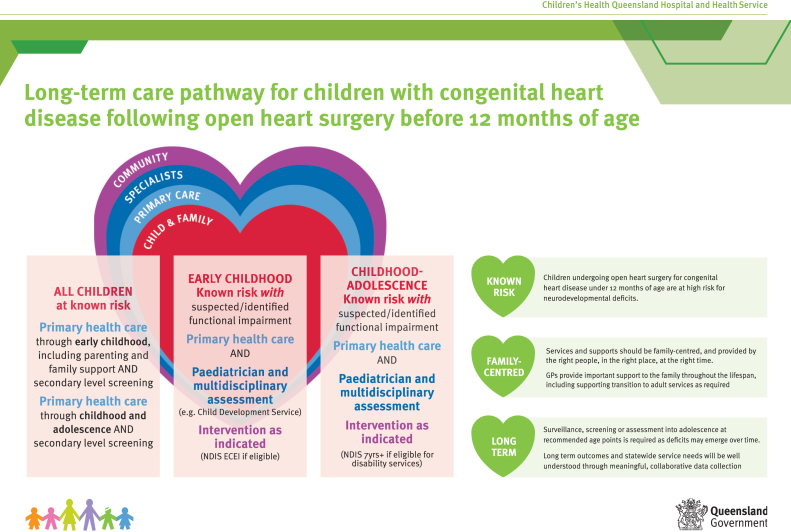
CHD LIFE cardiac developmental long-term care pathway. The pathway and companion document are available at https://www.childrens.health.qld.gov.au/wp-content/uploads/PDF/pink-book.pdf.^20^ CHD LIFE, Congenital Heart Disease Long-term Improvement in Functional hEalth; ECEI, Early Childhood Early Intervention; NDIS, National Disability Insurance Scheme.

Statewide partnerships with the Queensland Child and Youth Clinical Network and local and regional hospital and health services were established. A clinical service redesign methodology was employed with the aim of developing a sustainable and family-centred approach to developmental follow-up that contextualized AHA recommendations to the local health care context.^20^ The long-term developmental care pathway illustrated in Figure 1 and companion document (available at: https://www.childrens.health.qld.gov.au/wp-content/uploads/PDF/pink-book.pdf) provide follow-up recommendations. These recommendations recognize that there may be variability in availability of and eligibility and parental preferences for service access across statewide hospital and health jurisdictions. In addition to primary (general practitioner and child health) and hospital- and community-based developmental (general and developmental paediatric and allied health therapy) services, engagement and ongoing partnership with consumers including HeartKids, the national advocacy group for CHD in Australia, was an important element of the care pathway development. After initial care pathway implementation in 2018, a range of strategies have been adopted to support ongoing pathway engagement including targeted health service education, service referrals and electronic health record alerts, and ongoing parent education to support the understanding of developmental risk and follow-up recommendations. To date, there are 328 children prospectively on the care pathway with 274 consented to the programme database (>80% target) where outcome and service access data collected from routine clinical care will inform future care pathway evaluation and development.

## Long-term Developmental Follow-up for Children With Congenital Heart Disease—Canadian Context

The recently published paper from Bolduc et al.^21^ presents an environmental scan of the developmental follow-up practices in tertiary care centres performing paediatric open-heart surgery in Canada, as well as structural barriers to successful developmental follow-up. Similar to reports of US-based follow-up programmes, variability in current practices is identified in programme/pathway structure, service delivery modalities and eligibility, and timing and frequency of assessment. Referral processes for intervention are also variable, reflective of local service eligibility criteria. Identified barriers to service delivery are similar to the reported experiences of both US-based programmes and our own, including human and financial resources (with funding often reliant on research funding/donations) and geographical distance to the surgical centre.

This paper makes a valuable contribution to the literature, where in addition to the need and recommendations for systematic long-term developmental screening and assessment for children with CHD being recognized by participant centres, the further need to contextualize recommendations to the local health care context was identified. Strengths of the study include a mixed-methods and nationwide approach, with inclusion of all centres providing open-heart surgery, although the authors acknowledge limitations including the potential for bias through single source respondents from participating centres. Limited inclusion of nonsurgical cardiac centres and community providers was also identified with the need for further studies to enable evaluation of their knowledge and referral practices to inform the development of future policy recommendations. The authors identify the importance of parental mental health assessment as a component of developmental follow-up. It will also be essential to understand the experiences of families of children both eligible and ineligible for current follow-up pathways to identify preferences and barriers to care.

## Common Learnings

Common learnings are identified when considering these findings in relation to our Australian experience as there are similar geographical and public health care challenges in both countries. Primarily, there is an identified need to contextualize current AHA evaluation and management recommendations to local health care systems, acknowledging state/provincial differences and needs. In recognizing both limitations and benefits of the public health system, screening processes to identify those who would benefit from evaluation can be valuable, and the expertise of community-based practitioners also acknowledged. Consideration of more cost-effective surveillance approaches in response to the resource-intensive nature of developmental evaluation has been previously suggested.^22^ Within the United Kingdom, challenges to implementing resource-intensive approaches across the National Health Service have been identified. An early recognition tool for use by nondevelopmental practitioners to enable a referral care pathway across health sectors has been developed and validated, with how developmental follow-up can be best implemented long-term within the local context to be determined.^23^^,^^24^

Consistent with the Canadian experience, we also identify the ongoing work needed to ensure an improved understanding of current national neurodevelopmental follow-up care practices and to develop and implement national policy and standards. In partnership with the Australian Centre for Health Services Innovation, all Australian cardiac centres, neurodevelopmental experts, HeartKids and Children's Hospital Foundation, the CHD LIFE+ programme of research is underway to develop family-centred care models to support long-term neurodevelopment for children with CHD nationally (Medical Research Future Fund ARGCHDG0035). The current CHD LIFE model will be evaluated from a health economic, health care provider and family perspective, providing valuable evidence of the impact of such programmes. Current national and international neurodevelopmental care models and practices for children with CHD will be identified and inform the codesign of tailored models with both health services and consumers. Consistent with Canadian and US programmes, we anticipate that there may not be a “one size fits all” approach, with considerable geographical and health service variability in Australia. Importantly, this work will align with and be informed by in the National Standards of Care for Childhood Onset Heart Disease that is currently in development, with neurodevelopment and psychological care a priority.

Central to the development of the CHD LIFE programme has been the establishment of and value placed on partnership—between cardiology and hospital- and community-based developmental services, health care providers, and families, as well as clinical and academic partnerships. Local champions across disciplines and health services have also been vital to drive advocacy and awareness to support ongoing developmental service access and care. The growth of local, national, and international initiatives is encouraging, providing continued opportunities to share learnings, and to further improve neurodevelopmental follow-up and outcomes for all children with CHD and their families.
